# 

**Published:** 1871-01

**Authors:** 


### INDEX TO VOL. XII.

Acute Rheumatism, Tincture of Iron in.......... 38
Adulteration of Quinia, detection of with Salicine.......... 38
Air in Wounds Inocuous.......... 39
Alcohol, Effects of on the Human System—*By N. S. Davis, M.D*.......... 47
Annual Report of Surgeon General U. S. A.......... 55
American Medical Association.......... 58—185—242—360—498
Alcohol and Tobacco, Comparative Effects of on the Human System—
*By N. S. Davis, M. D*.......... 129—274
Alumni Association Chicago Medical College, Meeting of.......... 185
Atmospheric Germs and their Relation to Diseases—*By F. H. Davis, M.D.*, 193
Alumni Association of Chicago Medical College.......... 216
Acquired Syphilis—*By William Wilson, M. D.. etc*..........s 222
Amputation at the Hip Joint.......... 231
Albuminuric Retinitis.......... 253
Annual Report of the Necrologist of the Alumni Asso. Chicago Med. Col. 281
Anthrax and Furuncle of the Face.......... 311
Associa'n of Amer. Med. Editors, Proceedings of at 3d annual meeting, 318-347
American Medical Association, President Stille's Opening Address.......... 351
Alcohol, Death from in Cities.......... 385
Anterior Splints.......... 388
Æsculapian Society of the Wabash Valley.......... 362
Antiseptic Ligature as applied in the Operation for Varicocele—
*By Emile Steiger, M. D*.......... 469
American Journal Obstetrics and Diseases of Women and Children.......... 497
American Association for the Advancement of Sciences.......... 502
Asthma, Belladonna in.......... 103
Angina Pectoris, Pathology of.......... 170
Alcoholic Stimulants, should Physicians prescribe them—
*By A. Y. McCormick, M. D*.......... 472
Artificial Fecundation.......... 180
Albuminuria in Variola.......... 687

Barracks and Hospitals of the U. S. Army, a Report on.......... 121
Bone, Influence of the Diet on the Composition of.......... 168
Buboes, Rapid Case of.......... 171
Boston City Hospital, Medical and Surgical Report of.......... 181
Buccol Cancer, a Case of.......... 309
Bright's Disease, Cured by Ulcers.......... 313
Board of Health of the City of Chicago, Report of.......... 377
Belladonna, Poisoning from ; Recovery.......... 318
Belladonna Plaster.......... 576
Biliary Calculi, Passage of a large number and of unusual size.......... 605
British Medical Association, Papers Read at.......... 621
Blood Corpuscles, Passage of, through the Vessels.......... 686
Bromide of Sodium.......... 668

Cases from the Note Book of Robert Robson, M. D.......... 13
Chronic Hydrocephalus—*By Prof. N. S. Davis*.......... 21
Chloroform, Death from.......... 26—214
Chloroform, Death from, prevented by electricity.......... 40
Correction.......... 60
Catheterism of the Larynx.......... 62
Cancer of Testicle—By T. G. Williams, M. D.......... 98
Counter-Irritation—By J. E. Hendricks, M. D.......... 99
Chorea, Cardiac, murmurs in.......... 148
Climate of Nebraska, Cerebral and Bowel Affections, etc.—By Noble Holton, M. D.......... 142
Comments of W. H., Assistant Editor *Chicago Medical Journal*, upon Opium in Metro-Peritonitis, briefly reviewed.......... 150
Clinical Cases in Mercy Hospital, Medical wards service N. S. Davis, M. D.......... 107—109—256—208—286—341—594—664
Cæsarean Operation performed four times on the same person.......... 141
Chorea minor, epidemic of.......... 169
Cerebral Hemorrhage, hemiplegia from.......... 169
Chloral in Midwifery.......... 171
Classic Men and Classic Scenes in Europe.......... 173
Chloroform Deaths.......... 183
College Commencement Exercises.......... 184
Cauliflower, case of—*By E. R. Willard. M. D.*.......... 205
Change of Life, in Health and Disease.......... 232
Cancer, a Unique Case of—*By Norman Bridge, M. D.*.......... 456
Cataract, the Modern Operation for.......... 497
Cæsarian Section, sutures in the uterus after.......... 233
Commencement Chicago Medical College.......... 240
Chronic Catarrh.......... 244
Cod Civer Oil, to disguise the taste of.......... 244
Chloroform in Lahor.......... 253
Chloral Hydrate in Obstetrics.......... 307
Chloral in Tetanus.......... 312
Chloral for Sea Sickness.......... 314
Chloral in Cerebral Diseases.......... —
Chronic Leucorrhea, Incurability of.......... 315
Carbolic Acid in Vascular Keratitis—*By C. Hixson*.......... 339
Chemistry, General Medical & Pharmaceutical—*By John Attfield, Ch. D.*, 376
Correspondence of Senior Editor.......... 378
Carbolic Acid, Treatment in the London Hospital.......... 386
Chloral, Cases of Poisoning From.......... 386
Carbolic Acid, a report on the Medical uses of—*By N. S. Davis, M. D.*.......... 399
Clinical Cases, Surg. Wards, Mercy Hosp. service of *Prof. Andrews*. 430—601—666
Clinical Surgery in the the Royal Infirmary Glasgow, Scot.......... 449
Clinical notes—*By F. K. Bailey*.......... 474
Cundurango.......... 571
Cholera at Antwerp.......... 572
Clinical Reports, Mercy Hospital Opthalmological Wards, service of *Prof. Jones*.......... 502—669
Clinical Reports, Gynæcological Wards, service of *Prof. Byford*.......... 604—661
Climate of the Northwest and Nervous Diseases.......... 620
Chloral Hydrate, Effects of on the Blood.......... 635
Chloroform, the Element that Kills in.......... 613
Choroid Ossification of the—*By C. Hixson, M. D.*.......... 155
Compression Treatment of Aneurism.......... 696
Chronic Inflammation, etc., of the Unimpregnated Uterus, a Treatise on *By Prof. W. H. Byford*.......... 232
Cundurango—*By Edmund Andrews, M. D.*.......... 656

Dysentery, Ipecacuana in ........ 121
Diphtheria—*By J. J. McMullin, M. D* ........ 27
Degenerative Disease or Atrophy of the Gastric Tubules—
*By Austin Flint, M. D* ........ 111
Digitalis Leaves in Orchitis ........ 244
Diphtheria, Cauterization in ........ 249
Diseases of the Spine and of the Nerves—*By Chas. B. Radcliff, M. D* ........ 181
Diseases of Southern Indiana, Observations on—*By Rob. Robs n, M. D.* .... 199
Delirium Tremens, Treatment of ........ 247
Dysmenorrhœa ........ 697
Dislocation of Shoulder, New Method of Reducing ........ 315
Dermatological Conference ........ 314
Dysentery, Complicated with Malarious Fever ........ 461
Diseases of the Nervous System in Childhood—*By Charles West, M. D.* .... 570
Drunkenness, Increase of in England ........ 319
Diseases of the Nervous System, a Treatise on—*By Wm. A. Hammond, M. D.*, 497
Diarrhœ, Wet Sheets in ........ 693

Emetics, Administration of During Pregnancy, etc—*By J. G. Stokes, M. D.*, 1
Æsculapian Society of the Wabash Valley, Transactions of ........ 217
Endemics and the Soil ........ 309
Extraordinary Surgical Cases, a brief Sketch of a Few—
*By E. R. Willard, M. D* ........ 584
Excision of the Lateral half of the Tongue ........ 624
Explanation ........ 629
Enteric Fever, Spontaneous Origin of ........ 252
Ether, Death from Inhalation of ........ 254
Erysipelas, Sporadic and Epidemic—*By N. S. Davis, M.* D ........ 639

Functions and Disorders of the Reproductive Organs—*By Wm. Acton, M. R., C. S* ........ 527
Fibrous Structure of the Rectum Relieved by Incisions and Elastic Pressure ........ 226
Fractures and Dislocations—*By F. H. Hamilton, M. D., &c* ........ 628
Fætus Pushing of Through the Pelvis—*By S. S. Howley* ........ 101
Foreign Exchanges ........ 707
Fissure of Anus, Liniment for ........ 697

Gonorrhœa Produced by Chancre ........ 254
Graded Classes and Consecutive Study in Medical Colleges ........ 183
Good Appointment ........ 445
Gastic and Intestinal Tubules, the Pathological Relation of ........ 303
Ganglions, Treatment of ........ 119
Galvanization of the Sympathetic Observation on the Physiological and Therapeutical Effects—*By A. D. Rockwell, M. D* ........ 377

Hair as Sature and Ligature ........ 45
Heart Disease and Sea-Winds ........ 34
Hydrate Chloral, Large Dose—Recovery ........ 125
Hydrate Chloral—*By H. N. Hurlbut, M. D* ........ 216
Hiccough Cured by Chloral ........ 385
Health and Disease—A Physician's Counsel to Women in ........ 497
Horrors of War ........ 573
Homœopathy as it Was and Is—*By C. W. Earle, M. D* ........ 517
Headaches—*By H. G. Wright, M. D* ........ 628

Inflammation and Suppuration, Researches on ........ 684
Inflamed Testicles, Treatment of by Puncture ........ 171
Intussusception in an Infant Cured by Inflation of the Bowels ........ 36

Iodide of Lead Ointment........................ ............................. 120
Inverted Womb, Removal of.............................................................. 691
Intracranial Abscess, Cured by Trephining............ .................... 253
Illinois State Medical Society..........................................413—483—320—243
Iowa State Medical Society.............. ............................................ 384
Insanity..................................................................................... 385
Intra Uterine Injections................................. ........................... 385
Iodoform Ointment..................................................................697—386
Imposition .................................................................................. 448
Infections Ready, Mode of Preventing ................................................ 691
Injuries to Base of Brain, the Cause, Hæmorrhage, and Emphysema of the Lungs—*By C. E. Brown Sequard, M. D*............................ 494
Incision of the Elbow Joint to Relieve Anchyloais.................................. 621
Insanity and its Treatment.................... ....................................... 317
Inflammation of the Breast, Extract Conium in........................................ 690

Kine-Pock, Epidemic of........................................................................ 312

Letter from F. K. Bailey, M. D. ............................................................. 162
Letter from D. B. Trimble, M. D............................................................. 166
Letter from T. G. Williams, M. D............................................................ 346
Letter from G. W. Goodner, M. D., Ehrenbreitstein, Germany.................. 438
Lupus Exetens Treatment of—*E. Andrews, M. D*...................................... 589
Life Duration of..................................................................................... 385
Liquid Glass for Stiff Bandages in Fractures............................................ 166
Mortality Reports for Chicago............245—189—126—61—322—450—511—575—712
Mortality in Paris.................................................................................. —
Medical Society of the District of Columbia ............................................ 182
Money Receipts................ ..............................576—513—63—128—190
Medical College Commencement........ .................................................. 124
Muscular Exercise, Severe and Protracted, Effects of.................................. 502
Minnesota as a Resort for Consumptives..................................................... 46
Mechanical Anæsthetic.... .................................................................... 117
Medical Association of East Tennessee.................................................... 185
Morphia as a Parturient—*By George H. Vance, M. D*................................. 215
Medical Legislation.................... ................ ...... ............ 236
Medico-Legal Treatise.................. ..... ............................ 316
Medical and Surgical Memoirs—*By Joseph Jones, M. D*.............................. 377
Military Tract Medical Association.............................................................. 424
Morphia and Ergot as Parturients—*By George H. Vance, M. D*.................. 078
Minnesota State Medical Society................................... ........ 488
Medical Register and Directory of the United States.................................... 573
Midwifery a Manual of—*By Alfred Medows, M. D*.................................... 625
Medical Colleges in Chicago................................................................... 629
Medical Libraries Public......................................................................... 576
Morphiæ Sulph, as a Porturant—*By L. D. Robinson*.................................. 410
Mystery of Life, an Address—*By John M. Woodworth, M. D*........................ 257
Modern Therapeutics, a Compendium of.... ............................................. 317
Medical Profession. Mutual Relations of its Press and the Community, an Address—*By Horatio R. Storer, M. D*............ ................ .... 350
Muscalar Atrophy, Case of—*By V. Keysey, M. D*........................................ 608
Metro-Peritonitis—*By Teordore Griffin, M. D*........... ....................... 411
Mucus-Membrane, Transplantation of......................................................... 694
Microscopical Society, Illinois State.................... ............................ 709

Nutrition, Influence of Digestion on......................................................... 170
Note of Progress ....................................................................................... 448
Noble, Harrison M. D.—Obituary............................................................. 295
Noble, S. W.—Obituary.............................................................................. 295

Nitrogen, Effects of Severe and Protracted Muscular Exercise upon the Excretion of—*By A. Flint, Jr., M. D*.... 502
North Iowa Medical Society.... 486

Optholmology and Otology.... 60
Obituary.... 187
Oxygen Gas.... 246
Obstructive Dysmenorrhœa, an Operation for the Cure of.... 244
Opium and Belladonna, Antagonism Between—*By Lyman Ware, M. D*.... 608

Paralysis, Partial from Reflex Irritation.... 48
Pneumonia.... 118
Paracentisis Tympani—*By W. H. Fitch, M. D*.... 204
Prostitution and its Sanitary Management—*By E. Andrews, M. D*.... 65
Permonganate of Potassa.... 172
Postponement.... 244
Perforating Ulcer of Bladder.... 251
Pathological Relations of the Gastric and Intestinal Tubules—*By A. Flint, M. D*.... 303
Polypus of the Oesophogas—*By J. T. Everett, M. D*.... 453
Protracted and Complicated Disease, a Case of—*By L. D. Robinson, M. D*.... 546
Paris, Prussian Siege of.... 624
Publications, Notice of.... 639
Placenta Prævia, a Short History of Six Cases of—*By E. R. Willard, M.D*. 389
Preliminary Education.... 498
Phantom Abdominal Tumors, Diagnosis of.... 623
Pruritis and Prurigo, Administration of Carbolic Acid in.... 314
Prostitution, Regulation or Suppression of, the Legal Principles Involved—*By George Andrews, Esq*.... 334
Paralysis of Third Nerve, Two Cases of—*By C. K. Fiske, M. D*.... 688
Poisons, Varying Effects of on Different Animals.... 690

Rain Fall, with Comparative Mortality.... 147
Re-Fracture of Bones.... 120
Removal.... 244
Rare Cases—*By Alfred A. Castleman, M. D*.... 560
Relapsing Fever, Observations on, as it Occurred in Philadelphia in 1869-70.... 40
Revaccination, Importance of.... 315
Reflex Insanity, The Causation, Care and Treatment of.... 316
Report on Medical Education.... 671
Report on Otology—*By Samuel J. Jones, M. D*.... 680

Simpson, Dr., Cause of Death.... 37
Science vs. Senses—*By Justice*.... 30
Skin Grafting.... 42
Surgical Papers from Transactions of Amer. Med. Association.... 50
Stramonium Poisoning, Antidotal Power of Opium.... 30
Simple Dressing by Continued Moisture.... 35
Syphilis of Nervous System.... 44—33
Social Evil, The.... 124
Satan in Society—*By a Physician*.... 123
Scarlet Fever.... 119
Small Pox.... 119
Sin the Supposed Cause of Disease.... 118
Spermattorrhea, Its Causes and Symptoms—*By Robert Bartholow, A. M., M. D*.... 188
Sex of the Fœtus in Utero Determined by the Number of Cardiac Pulsations.... 188

Sympathetic Nervous Troubles from the Presence of Tenia................ 249
Scrofuloderma........................................................ 297
Specific Action of Tissues and their Elements—*By Daniel Lichty, M. D*.... 325
Spina Bifida ........................................................ 513
Scarlatina, Diseased Kidneys in—*By Carl Prœgler, M. D*................. 551
Syphilitic Paralysis—*By Lyman Ware, M. D*............................ 552
Synovitis, Acute; Incision into the Joint..............................449—523
Susquichloride of Iron in Rheumatism.................................. 695
Scalp Wound, Recovery from........................................... 172
Skimmed Milk as a Diet in Disease.................................... 229
Summer Course of Medical Instruction................................. 243
Small Pox in London.................................................. 254
Scarlet Fever, Extreme Contagiousness of............................. 117
Scarlet Fever, Treatment of........................................... 312
Subcutaneous Injections.............................................. 313
Small Pox, Treated with Sulphur Fumigations, etc ....................... 692

Tobacco—A Paper Read before the Chicago Medical Society—*By T. A. Emmons, M. D*.......................................................
Tabular History and Analysis—*By J. B. Upham, M. D*...................... 49
Transactions of Illinois State Medical Society, 1870..................... 57
Thoracentisis ........................................................ 250
Tongue, Rules in operating upon the.................................... 251
Tetanus. Chloral Hydrate in...........................................385--312
Tea Leaves as an Application to Burns—*by W. H. Searles*................. 207
Traumatic Paralysis of the Facial Nerve—*By Alfred E. Walker, M. D*..... 397
Typhus and Dysentery—*By Rudolph Virchow*................................ 207
Tetanus, Calabar Bean in............................................... 432
Therapeutics, Practical—*By E. J. Waring, M. D*.......................... 627
Turpentine, Therapeutical Action and Uses of............................ 612
Typho-Malarial Fever, with Symptoms of Cerobro-Spinal Irritation—*By N. S. Davis, M. D*.................................................. 588
Treatise on Localized Electrization—*By G. D. Duchenne*................... 626

Urea Found in the Liver ............................................... 58
Ulcers, Cure by a Recently Devised Operation............................ 311
Urine, Action of on the Tissues ......................................... 310
Ulcers, a Cause of Bright's Disease...................................... 31
Uræmic Convulsons, Bromide of Potassium—*By Edw. A. Carpenter, M. D.*, 652

Variola, Ten Days after Successful Vaccination........................... 97
Vaccination, Decline of in Great Britain................................. 385
Veratrum, Effects of a Large Dose of..................................... 471
Varicella in San Francisco............................................... 619

Wisconsin State Medical Society.......................................490—448—318
Woman's Hospital Medical College......................................... 184
When Doctors Differ, where is Safety...................................... 294
Wasting Diseases of Infants and Children.................................. 317
Women's and Children's Diseases, Handy-Book of—*By Eml. Dillenberger*, 626

